# Erratum to: Untested, unproven, and unethical: the promotion and provision of autologous stem cell therapies in Australia

**DOI:** 10.1186/s13287-016-0329-9

**Published:** 2016-05-05

**Authors:** Alison K. McLean, Cameron Stewart, Ian Kerridge

**Affiliations:** Sydney Medical School, Edward Ford Building (A27), University of Sydney, Sydney, Australia; Centre for Values, Ethics and the Law in Medicine, K25, Medical Foundation Building, Sydney Medical School, University of Sydney, Sydney, Australia; Haematology Department, Royal North Shore Hospital, Sydney, Australia; Northern Blood Research Centre, Kolling Institute, Sydney, Australia

## Erratum

Due to an error during the production process, this article was unintentionally published twice in this journal [1,2] with the following citations and DOIs:

- McLean AK, Stewart C and Kerridge I: **Untested, unproven, and unethical: the promotion and provision of autologous stem cell therapies in Australia.***Stem Cell Res Ther* 2015, **6**:12, DOI: 10.1186/scrt543
- McLean AK, Stewart C and Kerridge I: **Untested, unproven, and unethical: the promotion and provision of autologous stem cell therapies in Australia.***Stem Cell Res Ther* 2015, **6**:33, DOI: 10.1186/s13287-015-0047-8

The version McLean AK, Stewart C and Kerridge I: **Untested, unproven, and unethical: the promotion and provision of autologous stem cell therapies in Australia.***Stem Cell Res Ther* 2015, **6**:12, DOI: 10.1186/scrt543 is to be ignored, and the original article should be cited as:

- McLean AK, Stewart C and Kerridge I: **Untested, unproven, and unethical: the promotion and provision of autologous stem cell therapies in Australia.***Stem Cell Res Ther* 2015, **6**:33, DOI: 10.1186/s13287-015-0047-8

The Publisher is issuing this Erratum to alert readers to this error and to confirm the correct citation details and DOI for this article. We apologize for any inconvenience caused.
